# A signature for immune response correlates with HCV treatment outcome in Caucasian subjects

**DOI:** 10.1016/j.dib.2015.01.009

**Published:** 2015-02-11

**Authors:** Brian J. Hare, Eric Haseltine, Mark Fleming, Daniel Chelsky, Laura McIntosh, Rene Allard, Martyn Botfield

**Affiliations:** a Vertex Pharmaceuticals Incorporated, Boston, MA, USA; b Caprion Proteomics, Montreal, Quebec, Canada H2X 3Y7

## Abstract

This data article contains Supplementary material for a published research article describing a whole-blood proteomic signature that predicts treatment outcome for subjects infected with hepatitis C virus (HCV) [1]. The proteomic signature is derived from whole-blood samples from subjects infected with HCV. The article includes detailed experimental and computational methods used in the analysis. The article also includes tables of demographic and other information about the subjects. Finally, the article includes several figures and tables showing detailed results of the analyses (e.g. lists of identified proteins and coefficients/ROC curves for the regression models).

## Specifications table

**Table:** 

Subject area	Biology
More specific subject area	Hepatitis C
Type of data	Tables, figures, methods
How data was acquired	Mass spectroscopy: Waters QToF Ultima, AB Sciex QTRAP 5500
Data format	Analyzed
Experimental factors	Samples were depleted of abundant proteins and digested with trypsin
Experimental features	Liquid chromatography–mass spectrometry and multiple reaction monitoring analyses of plasma samples collected from subjects with HCV prior to treatment
Data source location	Clinical sites in United States and Europe
Data accessibility	All publicly released data is within this article

## Value of the data

• This study is one of only two published broad proteomic profiling studies of HCV patients.
• The signature predicting outcome to HCV treatment is unique and warrants follow-up by other investigators.
• The signature provides insight into biological mechanisms underlying response to HCV treatment.

## 1 Supplementary methods

### 1.1 Sample preparation and mass spectrometry

Samples were depleted of abundant proteins with two sequential antibody columns (IgY14 and Supermix, Sigma) and the remaining lower abundance proteins were digested with trypsin. Each sample was then further fractionated by reversed phase liquid chromatography, coupled by electrospray to a Waters QTOF mass spectrometer (LC–MS). In this analysis, 8374 chromatrographic components were detected and quantified.

Tandem mass spectrometry (LC–MS/MS) was subsequently used to identify the proteins from which differentially-expressed peptides between treatment responders and non-responders were derived. Seven samples were omitted from the statistical analysis because they appeared to contain very high abundance proteins that were not completely removed by the immunoaffinity depletion.

### 1.2 Data analysis

Detected chromatographic component ions were matched across all samples and compared for relative peak intensity. Peak intensity was normalized to account for small differences in protein concentration between samples. A multifactor ANOVA analysis was then applied to identify components that were differentially expressed between the groups of interest. High stringency thresholds were used to ensure the statistical significance of the identified components. Details of these steps are provided below.

Seven samples were omitted from the statistical analysis. These samples appeared to contain very high abundance proteins that were not completely removed by the immunoaffinity depletion.

#### 1.2.1 Normalization

All intensity values are log (base *e*) transformed with values less than zero were replaced by zero. The sum of the intensities for each sample is then calculated. In this study, samples lying between the 25th and 75th percentiles were used to create an average sample (i.e. the reference sample), against which the actual samples were then normalized. The normalization factors are chosen in such a way that the log ratios between the actual and Reference samples over all the components is adjusted to 0.

#### 1.2.2 Statistical analysis

An ANOVA was used to determine the differentially-expressed components for various comparisons defined in the study objectives. The FDR and *q*-values are calculated based on the *p*-values obtained from the ANOVA, using Storey׳s method to make multiple testing adjustments (implemented in MATLAB). Thresholds used to determine which components were differentially expressed within the various comparisons consisted of a differential expression of at least 1.8 (ANOVA), a maximum *q*-value of 0.1 (Storey׳s method), a component intensity ≥70 in at least 5 samples and a charge ≥2.

#### 1.2.3 Predictive model

Decline in viral titer at week 4 was used as the outcome in the model because it is a continuous variable and correlates well with the clinically-accepted outcome of sustained viral response (SVR). All of the normalized components were used in an elastic-net regularized linear regression model [8] to predict change-from-baseline viral titer at week 4 (using log 10 transformed viral titers) using R 3.0.1 (R Core Team 2013. R: A language and environment for statistical computing. R Foundation for Statistical Computing, Vienna, Austria. URL http://www.R-project.org/). The performance of the model was estimated using 10-fold cross validation with root mean square error used as the loss function. Both alpha and lambda were optimized using the caret library in R. ROC curves, AUC and sensitivity and specificity were computed using the AUC library in R.

#### 1.2.4 Sequencing and protein identification

The components found to be differentially expressed in the statistical analyses together with an additional 31 components that were best predictors of PR outcome based on a logistic regression model were targeted for sequencing on a Waters QTOF mass spectrometer and the resulting fragmentation patterns were matched to the corresponding peptide sequences found in a database composed of the IPI (International Protein Index) human proteins (version 3.68) and the NCBI HCV genotype 1 database (taxonomy ID 41865). Two distinct N-glycosylated forms of an LGALS3BP peptide (AAIPSALDTNSSK) were identified. The two glycans have primary structures Hex5HexHAc4NeuAc1 and Hex5HexHAc4NeuAc2. Two isoforms of parvin, PARVA and PARVB, are not uniquely distinguished by component peptides and are so are denoted PARVA/B.

The final assay consisted of 176 peptides and a total of 354 transitions, including both peptide precursor and fragment ion mass-to-charge ratios, monitored by the mass spectrometer

## 2 MRM data

### 2.1 Sample preparation and MRM analysis

Samples were randomly assigned to three processing batches and were depleted of high and medium abundance proteins using three matching columns, consisting of a single batch of mixed IgY14 and Supermix resins (Sigma). The unbound fraction of each sample was digested with trypsin and desalted. The samples were dried by vacuum evaporation and stored at 20 °C in a sealed 96-well injection plate.

For the MRM analysis, samples were resolubilized in 10 μL of a solution containing 95/5 water/acetronitile, 0.2% formic acid and 250 ng/ml of four internal standard peptides with the amino acid sequences ASSILAT, FSDISAAK, NVDQSLLELHK and QNNGAFDETLFR. A total of 354 transitions were monitored, corresponding to 176 peptides, representing 71 proteins that were identified as differentially expressed in the discovery stage. The MRM assay was developed with the help of 176 synthetic reference petides, corresponding in sequence to the peptides of interest. The peptide transition peak areas were detected and integrated using Elucidator software (Rosetta Biosciences) in combination with software developed at Caprion for the automated MRM peak integration workflow. The peak area of each monitored transition was used as input for the statistical analysis. Eight samples were removed from the analysis set because of apparent hemolysis or low intensity data.

### 2.2 Data analysis

#### 2.2.1 Normalization

The raw intensities (*I*) were transformed using the function *l*=Ln(*I*+1) where Ln is the natural log function. For each sample, all peptide transition peak areas were normalized against the average of two transitions of the internal standard peptide (FSDIAAK). A linear prediction model was then fitted, lfit=mu+*b*⁎*R* where mu is a constant background. *R* is the “reference transition” and lfit is the predicted intensity of the transition to normalize. As a final step, the normalization was performed, as *I*=*I*−lfit+mean (*I*). The detection rate (DR) was defined as the proportion of samples with a raw value (i.e. untransformed and non-normalized) above the limit of quantification which was determined to be 10,000. Transitions were only included in the analysis set if they had detection rates greater than 25% in each of three subgroups of subjects: treatment responder, treatment non-responders and healthy controls. A total of 195 transitions representing 54 proteins were included in the analysis set.

#### 2.2.2 Calculation of protein intensities from component peptides

For each protein, transitions that passed the DR filtering criteria were combined together to create a “representative” protein. Component intensities in the log (base 2) scale were utilized. To combine the transitions, the following algorithm was applied:

1. Let *I*_p_ represent a matrix w/rows as samples and columns as transitions derived from a single protein.
2. Compute the covariance matrix of *I*_p_ and call it *Z*.
3. Decompose *Z* as *Z*=*QDQ*^t^ where *Q*^t^ is the transpose of matrix *Q*.
4. Let *d* be the column vector corresponding to the diagonal of *D* and *l*=trace(*d*). l is the sum of the eigenvalues.
5. Compute the protein intensity as *I*_r_=abs(*I*_p_*Qd*/*l*).

For proteins only represented by one transition in the analysis set (CLIC1, FERMT3, PARVA/B, FCGR2B and CTSD), the normalized intensity values for the transition were used directly as the protein intensities.

All transitions corresponding to the LGALS3BP protein were used together in the matrix *I*_p_ to calculate the LGALS3BP protein intensity since both glycosylated and unglycosylated components are strongly weighted in the first principal component of the eigenvector decomposition of the LGALS3BP covariance matrix. The glyocosylated peptides have weightings in the first principal component that rank 3,4,5 and 8 out of 22 total components and the first principal component accounts for 68% of the variability of LGALS3BP expression across the analysis set.

#### 2.2.3 Calculation of differential intensity ratios (DI*)*

The DI calculation was based on normalized intensities, but the data was transformed back to the original linear scale using the transformation ln=exp(ln)−1 before calculating DI. Raw intensity zero values were excluded from the DI calculations. The DI was defined as the ratio of the two median intensities.

#### 2.2.4 Univariate analyses

Statistical analyses were conducted using R (2.15.1). The Wilcoxon rank sum test was used to assess statistical significance of differences in protein intensities between groups. Multiple testing was controlled separately for each analysis using the Benjamini Hochberg procedure [9].

#### 2.2.5 Predictive model

The same methods that were used to build the predictive model in the discovery phase were used for the MRM data except that in addition to all of the normalized transitions the covariates body mass index, age, gender, fibrosis and baseline HCV RNA were also included in the feature set. Six subjects were excluded from the analyses because their week 4 HCV RNA value was missing. The performance of the model was estimated using 10-fold cross validation with misclassification error used as the loss function. Cross validation may overestimate the performance of the final model since some of the samples used in discovery stage were used again in the MRM analysis.

## 3 Data

Supplementary Fig. 1 is a Venn diagram showing differentially-expressed peptides between treatment responders and non-responders in the discovery stage for subjects treated with PR (peg-interferon/ribavirin) and T/PR (telaprevir/peg-interferon/ribavirin). Differentially-expressed peptides are defined as those with false discovery rate less than 0.05 and fold differential expression greater than 2. The overlap between the two treatments is highly statistically significant (*p*<2×10^−16^). Supplementary Fig. 2 shows ROC curves for predicting SVR for PR (A) and T/PR (B) subjects using only the five identified peptides as predictors. The model was fitted using only data from the PR subjects and is independently validated on the T/PR subjects. AUCs for A and B are 0.97 and 0.81, respectively. Supplementary Fig. 3 shows the ROC curve for predicting SVR for PR subjects in using MRM data. The AUC for this curve is 0.8. Supplementary Fig. 4 is a comparison of PR week 4 viral declines for the (A) discovery and (B) MRM analysis stages.

Supplementary Table 1 lists subject characteristics according to treatment group and response (SVR). Supplementary Table 2 lists oefficients in the model predicting treatment outcome from discovery phase. Supplementary Table 3 lists the clinical trials from which the samples used in the proteomic analyses were obtained. Supplementary Table 4 lists the numbers of differentially-expressed components identified from statistical comparisons in the discovery stage. Supplementary Table 5 lists the gene symbols for the proteins identified in the discovery stage. Supplementary Table 6 lists the coefficients in the model predicting treatment outcome from discovery stage data using only components that are identified. Supplementary Table 7 lists references for liver expression of the proteins that are significantly-differentially expressed between responders to PR. Supplementary Table 8 lists pairwise pearson correlations among differentially-expressed proteins and other clinical covariates. Supplementary Table 9 [2–7,10–12] are the references for Fig. 4 in [1].
