# Supplementary material for: A signature for immune response correlates with HCV treatment outcome in Caucasian subjects
Source: Data Brief. 2015 Feb 11;3:56–61. doi: 10.1016/j.dib.2015.01.009 (PMC4510051; doi:10.1016/j.dib.2015.01.009)
Supplement: Supplementary file 1 — Supplementary data [file mmc1.zip › supp_fig2.pptx]

## Slide 1
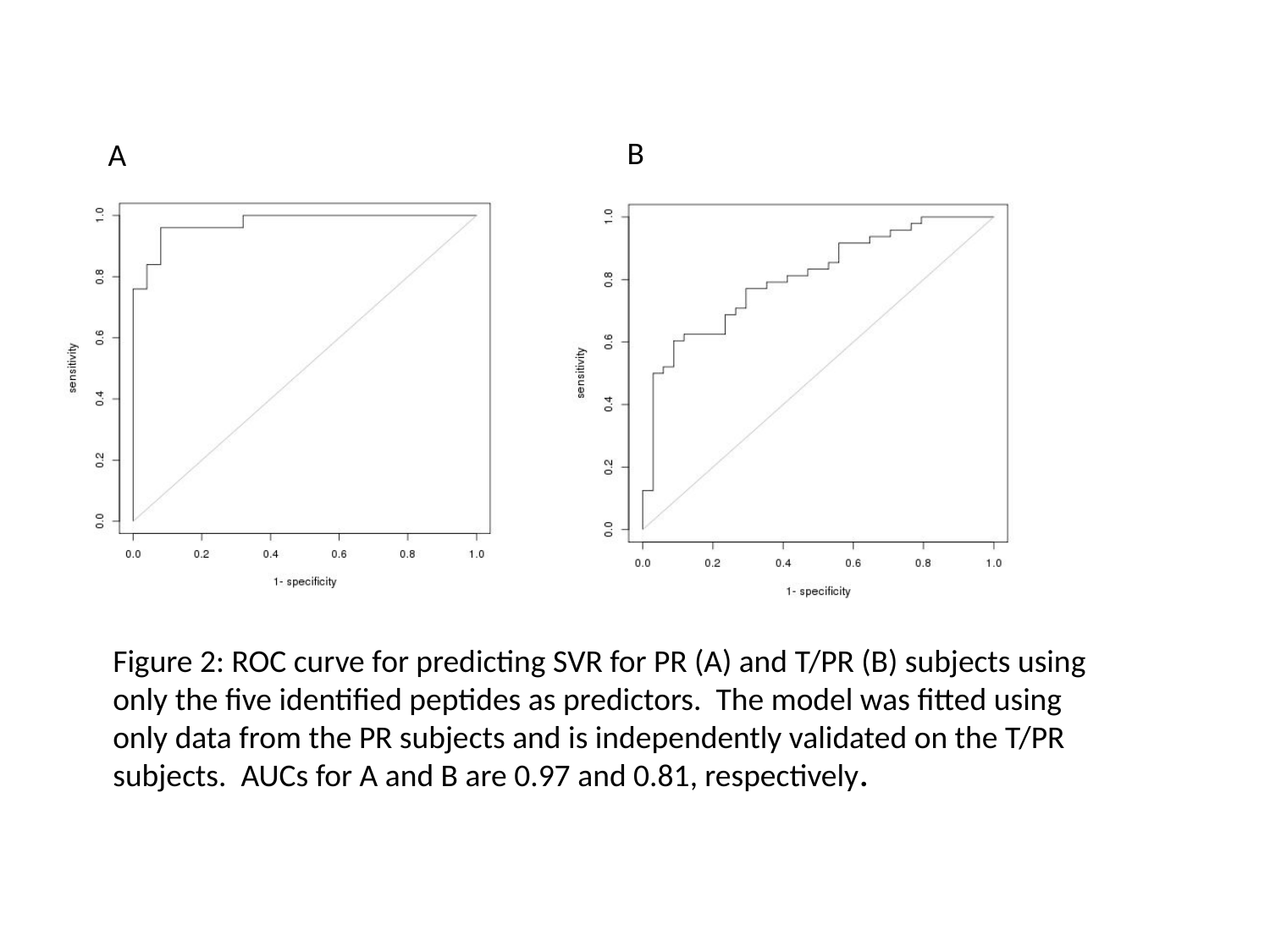

B
A
Figure 2: ROC curve for predicting SVR for PR (A) and T/PR (B) subjects using only the five identified peptides as predictors. The model was fitted using only data from the PR subjects and is independently validated on the T/PR subjects. AUCs for A and B are 0.97 and 0.81, respectively.
