# Supplementary material for: A signature for immune response correlates with HCV treatment outcome in Caucasian subjects
Source: Data Brief. 2015 Feb 11;3:56–61. doi: 10.1016/j.dib.2015.01.009 (PMC4510051; doi:10.1016/j.dib.2015.01.009)
Supplement: Supplementary file 1 — Supplementary data [file mmc1.zip › supp_table1.docx]

Supplementary Table 1: Subject characteristics according to treatment group and response (SVR)

| **Characteristic** | **PR** | | **T/PR (naive)** | | **T/PR (prior NR)** | |
| --- | --- | --- | --- | --- | --- | --- |
|  | SVR- | SVR+ | SVR- | SVR+ | SVR- | SVR+ |
| *Discovery stage* |  |  |  |  |  |  |
| N | 25 | 25 | 19 | 28 | 33 | 35 |
| Age – median (range) | 50 (28-59) | 45 (25-58) | 48 (22-56) | 47 (33-61) | 50 (36-61) | 51 (24-60) |
| Male, n (%) | 17 (68) | 14 (56) | 11 (58) | 15 (54) | 25 (76) | 20 (57) |
| Race or ethnic group – no. (%) |  |  |  |  |  |  |
| White | 23 (92) | 23 (92) | 7 (37) | 19 (68) | 26 (79) | 28 (80) |
| Black | 0 (0) | 0 (0) | 11 (58) | 8 (29) | 7 (21) | 7 (20) |
| Asian | 1 (4) | 1 (4) | 0 (0) | 0 (0) | 0(0) | (0) |
| Hispanic | 1 (4) | 1 (4) | 0(0) | 0(0) | 0(0) | 0(0) |
| Other | 0 (0) | 0 (0) | 1(0) | 1(0) | 0(0) | 0(0) |
| BMI – median (range) | 24.1(18.4-37.4 | 24.6(21.2–31.8) | 26.8(20.4-44.0) | 26.3(19.7-40.9) | 26.5(20.3-44.4) | 26.8(19.7-40.4) |
| Fibrosis on previous liver biopsy – no (%) |  |  |  |  |  |  |
| None or minimal | 8 (32) | 7 (28) | 5 (26) | 11 (39) | 8 (24) | 8 (23) |
| Portal | 10 (40) | 15 (60) | 11 (58) | 11 (39) | 8 (24) | 19( 54) |
| Bridging | 7 (28) | 3 (12) | 3 (16) | 6 (21) | 13 (39) | 5 (14) |
| Cirrhosis | 0 (0) | 0 (0) | 0 (0) | 0 (0) | 4 (12) | 3 (9) |
| Baseline HCV RNA – log10 IU/ml | 6.8 +/- 6.7 | 6.5 +/- 6.7 | 6.8 +/- 6.8 | 6.9 +/- 6.8 | 7.1 +/- 7.1 | 6.9 +/- 6.9 |
| HCV RNA >= 800,000 IU/ml – no. (%) | 25 (100) | 14 (56) | 15 (79) | 26 (93) | 32 (97) | 33 (94) |
| ΔHCV RNA titer (week 4 vs. baseline) – median (range) log10 IU/ml | 0.92 (0.15 – 2.0) | 4.7 (4.1 – 6.1) | 5.0 (0.87 – 6.6) | 6.2 (4.1 – 6.7) | 4.4 (0.7 – 6.8) | 5.8 (3.9 – 6.9) |
| HCV genotype 1 subtype – no. (%) |  |  |  |  |  |  |
| 1a | 14 (56) | 13 (52) | 11 (58) | 14 (50) | 23 (70) | 23 (66) |
| 1b | 10 (40) | 8 (32) | 5 (26) | 12 (43) | 8 (24) | 4 (11) |
| Other or indeterminate | 1 (4) | 4 (16) | 3 (16) | 2 (7) | 2 (6) | 8 (23) |
| *Samples analyzed w/MRM assays* |  |  |  |  |  |  |
| N | 88 | 68 |  |  |  |  |
| Age – median (range) | 50(24-64) | 44 (18-64) |  |  |  |  |
| Race or ethnic group – no (%) |  |  |  |  |  |  |
| White | 72 (82) | 62 (91) |  |  |  |  |
| Black | 10 (11) | 1 (1) |  |  |  |  |
| Asian | 2 (2) | 2 (3) |  |  |  |  |
| Hispanic | 4 (5) | 2 (3) |  |  |  |  |
| Other | 0 (0) | 1 (1) |  |  |  |  |
| Male, n (%) | 50 (57) | 39 (57) |  |  |  |  |
| BMI | 25.3(18.4-37.4) | 24.8(17.3-37.6) |  |  |  |  |
| Fibrosis |  |  |  |  |  |  |
| No or minimal | 23 (26) | 24 (35) |  |  |  |  |
| Portal | 46 (52) | 36 (53) |  |  |  |  |
| Bridging | 19 (22) | 8 (12) |  |  |  |  |
| Cirrhosis | 0 (0) | 0 (0) |  |  |  |  |
| HCV RNA – log10 IU/ml | 6.8 +/- 6.7 | 6.8 +/- 6.9 |  |  |  |  |
| HCV RNA >= 800,000 IU/ml – no. (%) | 84 (95) | 52 (76) |  |  |  |  |
