# Supplementary material for: A signature for immune response correlates with HCV treatment outcome in Caucasian subjects
Source: Data Brief. 2015 Feb 11;3:56–61. doi: 10.1016/j.dib.2015.01.009 (PMC4510051; doi:10.1016/j.dib.2015.01.009)
Supplement: Supplementary file 1 — Supplementary data [file mmc1.zip › supp_table2.docx]

Supplementray Table 2: Coefficients in model predicting treatment outcome from discovery phase

| Transition id | coefficient | Peptide sequence | glycan | protein |
| --- | --- | --- | --- | --- |
| 7919386 | 0.388 |  |  |  |
| 7919767 | -0.0243 | TLQALEFHTVPF |  | LGALS3BP |
| 7920516 | 0.279 |  |  |  |
| 7920466 | -0.0111 |  |  |  |
| 7921271 | -0.298 |  |  |  |
| 7922733 | 0.167 | YPSLSIHGIEGAFDEPGTK |  | CNDP1 |
| 7927038 | -0.0258 |  |  |  |
| 7930060 | -0.0183 |  |  |  |
| 7931054 | -0.0214 | ELSEALGQIFDSQR |  | LGALS3BP |
| 7932784 | 0.1119 |  |  |  |
| 7934066 | 0.00912 |  |  |  |
| 7935538 | -0.165 | Hex5HexNAc4NeuAc2 | AAIPSADLTNSSK | LGALS3BP |
| 7936038 | -0.116 | Hex5HexNAc4NeuAc1 | AAIPSALDTNSSK | LGALS3BP |
|  |  |  |  |  |
| Intercept | -1.93 |  |  |  |
