# Supplementary material for: A signature for immune response correlates with HCV treatment outcome in Caucasian subjects
Source: Data Brief. 2015 Feb 11;3:56–61. doi: 10.1016/j.dib.2015.01.009 (PMC4510051; doi:10.1016/j.dib.2015.01.009)
Supplement: Supplementary file 1 — Supplementary data [file mmc1.zip › supp_table3.docx]

Supplementary Table 3: Study Samples

| Group | Treatment | Race | Study | Response (SVR) | # samples |
| --- | --- | --- | --- | --- | --- |
| A | PR | Caucasian, Asian, Hispanic | PROVE 1 & 2 | - | 25 |
| B | PR | Caucasian, Asian, Hispanic | PROVE 1 & 2 | + | 25 |
| C1 | T/PR | Caucasian | PROVE 1 & 2 | - | 9 |
| C2 | T/PR | Caucasian | PROVE 3 | - | 29 |
| D1 | T/PR | Caucasian | PROVE 1 & 2 | + | 20 |
| D2 | T/PR | Caucasian | PROVE 3 | + | 29 |
| E1 | T/PR | African American | PROVE 1 & 2 | - | 11 |
| E2 | T/PR | African American | PROVE 3 | - | 8 |
| E3 | T/PR | African American | PROVE 1 & 2 | + | 8 |
| E4 | T/PR | African American | PROVE 3 | + | 8 |
