# Supplementary material for: A signature for immune response correlates with HCV treatment outcome in Caucasian subjects
Source: Data Brief. 2015 Feb 11;3:56–61. doi: 10.1016/j.dib.2015.01.009 (PMC4510051; doi:10.1016/j.dib.2015.01.009)
Supplement: Supplementary file 1 — Supplementary data [file mmc1.zip › supp_table4.docx]

Supplementary Table 4: Number of differentially-expressed components from statistical comparisons

| Comparison* | # of differentially-expressed components** |
| --- | --- |
| D1 vs C1 | 32 |
| D2 vs C2 | 71 |
| (D1,D2) vs (C1, C2) | 558 |
| (D1,D2,E3,D4) vs (C1,C2,E1,E2) | 109 |
| B vs A | 423 |
| (D1,D2) vs B | 5 (188) |
| D1 vs B | 0 (0) |
| D2 vs B | 561 (675) |
| (E1,E2) vs (C1, C2) | 435 |
| (E3, E4) vs (D1, D2) | 25 |
| (E3, E4) vs (E1, E2) | 0 |
| (B1,D1,D2,E3,E4) vs (A,C1,C2,E1,E2) | 219 |
| (B,D1,D2) vs (A,C1,C2) | 419 |

*Groups are defined in Supplementary Table 3

**Differentially expressed components have fold change > 1.8 and q < 0.1. Numbers of differentially expressed components found between parentheses were obtained with a more relaxed q-value < 0.4.
