# Supplementary material for: A signature for immune response correlates with HCV treatment outcome in Caucasian subjects
Source: Data Brief. 2015 Feb 11;3:56–61. doi: 10.1016/j.dib.2015.01.009 (PMC4510051; doi:10.1016/j.dib.2015.01.009)
Supplement: Supplementary file 1 — Supplementary data [file mmc1.zip › supp_table5.docx]

Supplementary Table 5: Proteins identified in discovery stage

| LPA | CLIC1 | APOB | MMRN1 | PON1 |
| --- | --- | --- | --- | --- |
| CNDP1 | F13A1 | A2M | APOE | C2 |
| TPM4 | VCL | SERPINA1 | MBL2 | BTD |
| GAPDH | FLNA | LGALS3BP | ICAM1 | APOA4 |
| FKBP1A | SDPR | CTSD | CD163 | AZGP1 |
| PARVA/B | TAGLN2 | FTL | IGFALS | SERPINF1 |
| VCP | C9 | CHI3L1 | CPN2 | C1RL |
| PPIA | CP | FCGBP | APCS | SERPINA7 |
| PFN1 | YWHAE | CD5L | BCHE | CFI |
| CAP1 | ORM1 | KRT9 | APOC3 | SERPINA6 |
| ILK | HPR | HBB | HABP2 | FCGR2B |
| PLEK | FERMT3 | SERPIND1 | HGFAC |  |
| GSTP1 | PZP | FGB | ALB |  |
| TLN1 | APOC4 | FGA | PGLYRP2 |  |
| ZYX | CLEC3B | SEPP1 | TF |  |
