# Supplementary material for: A signature for immune response correlates with HCV treatment outcome in Caucasian subjects
Source: Data Brief. 2015 Feb 11;3:56–61. doi: 10.1016/j.dib.2015.01.009 (PMC4510051; doi:10.1016/j.dib.2015.01.009)
Supplement: Supplementary file 1 — Supplementary data [file mmc1.zip › supp_table6.docx]

Supplementary Table 6: Coefficients in model predicting treatment outcome from discovery phase using only identified components

| Transition id | coefficient | Peptide sequence | glycan | protein |
| --- | --- | --- | --- | --- |
| 7919767 | 0.682 | TLQALEFHTVPF |  | LGALS3BP |
| 7922733 | 0.813 | YPSLSIHGIEGAFDEPGTK |  | CNDP1 |
| 7931054 | -0.0916 | ELSEALGQIFDSQR |  | LGALS3BP |
| 7935538 | 0.0789 | Hex5HexNAc4NeuAc2 | AAIPSADLTNSSK | LGALS3BP |
| 7936038 | -0.588 | Hex5HexNAc4NeuAc1 | AAIPSALDTNSSK | LGALS3BP |
|  |  |  |  |  |
| Intercept | -7.33 |  |  |  |
