# Supplementary material for: A signature for immune response correlates with HCV treatment outcome in Caucasian subjects
Source: Data Brief. 2015 Feb 11;3:56–61. doi: 10.1016/j.dib.2015.01.009 (PMC4510051; doi:10.1016/j.dib.2015.01.009)
Supplement: Supplementary file 1 — Supplementary data [file mmc1.zip › supp_table7.docx]

Supplementary Table 7: Liver expression of proteins in response signature

|  | Expression in liver | | | |
| --- | --- | --- | --- | --- |
| Protein | Literature | Reference(pmid) | HUPO liver | Protein Atlas |
| FERMT3 | Nothing relevant found |  | Yes | Pending |
| PARVA/B | Integrin-linked kinase is involved in matrix-induced hepatocyte differentiation | 17194454 | Yes | Strong |
| CNDP1 | A hepatic peptidase that decreases in subjects with cirrhosis and hepatoma | 3731453 | No | Weak |
| PFN1 | Upregulation of PFN1 is associated with hepatocellular carcinoma in human (potential biomarker). | 16335951 | Yes | Moderate |
| ZYX | Novel identification of zyxin upregulations in the motile phenotype of hepatocellular carcinoma | 16680155 | Yes | None (but weak to moderate in liver cancer) |
| TLN1 | Talin1 is possibly involved in the process of the carcinogenesis, infiltration and metastasis of HCC and has potential as a marker for diagnosis and prognostic assessment | 22471464 | Yes | Low |
| TAGLN2 | A novel interplay between oncogenic PFTK1 protein kinase and tumor suppressor TAGLN2 in the control of liver cancer cell motility | 21577206 | Yes | Low |
| APOA4 | Expression of APOA4 in human liver by transcriptional profiling | 18550803 | Yes | Moderate |
| VCL | Hepatic stellate cells (HSCs) have important roles in the pathogenesis of liver fibrosis and cirrhosis; Vinculin was suggested as HSC marker | 19052772 | Yes | Moderate |
| AZGP1 | Decreased expression of zinc-alpha2-glycoprotein in hepatocellular carcinoma associates with poor prognosis | 22625427 | Yes | Moderate |
| CLIC1 | CLIC1 is upregulated in human cells by HCV polyprotein expression from a viral vector | PMC2553408 | Yes | Weak |
| C9 | Downregulation of human C9 mRNA in hepatocellular carcinoma is associated with hepatocellular carcinoma in human | 19686584 | Yes | Weak |
| ORM1 | Expression of human ORM1 mRNA in hepatoblastoma expressing human AFP protein is associated with liver cancer in human. | 15221005 | Yes | Strong |
| GSTP1 | Upregulation of human GSTP1 protein in liver is associated with cirrhosis in human | 8770536 | Yes | Moderate |
