# Supplementary material for: A signature for immune response correlates with HCV treatment outcome in Caucasian subjects
Source: Data Brief. 2015 Feb 11;3:56–61. doi: 10.1016/j.dib.2015.01.009 (PMC4510051; doi:10.1016/j.dib.2015.01.009)
Supplement: Supplementary file 1 — Supplementary data [file mmc1.zip › supp_table8.docx]

Supplementary Table 8: Pairwise pearson correlations among differentially-expressed proteins and other clinical covariates

|  | CLIC1 | ZYX | TAGLN2 | PFN1 | FERMT3 | PARVA/B | GSTP1 | VCL | TLN1 | APOA4 |
| --- | --- | --- | --- | --- | --- | --- | --- | --- | --- | --- |
| CLIC1 | 1.00 | 0.80 | 0.76 | 0.74 | 0.71 | 0.63 | 0.71 | 0.72 | 0.66 | -0.01 |
| ZYX | 0.80 | 1.00 | 0.91 | 0.88 | 0.80 | 0.71 | 0.72 | 0.82 | 0.77 | -0.08 |
| TAGLN2 | 0.76 | 0.91 | 1.00 | 0.89 | 0.81 | 0.70 | 0.75 | 0.84 | 0.77 | 0.05 |
| PFN1 | 0.74 | 0.88 | 0.89 | 1.00 | 0.75 | 0.72 | 0.76 | 0.89 | 0.80 | 0.06 |
| FERMT3 | 0.71 | 0.80 | 0.81 | 0.75 | 1.00 | 0.76 | 0.75 | 0.74 | 0.73 | 0.13 |
| PARVA/B | 0.63 | 0.71 | 0.70 | 0.72 | 0.76 | 1.00 | 0.74 | 0.78 | 0.79 | 0.14 |
| GSTP1 | 0.71 | 0.72 | 0.75 | 0.76 | 0.75 | 0.74 | 1.00 | 0.79 | 0.81 | 0.16 |
| VCL | 0.72 | 0.82 | 0.84 | 0.89 | 0.74 | 0.78 | 0.79 | 1.00 | 0.86 | 0.08 |
| TLN1 | 0.66 | 0.77 | 0.77 | 0.80 | 0.73 | 0.79 | 0.81 | 0.86 | 1.00 | 0.21 |
| APOA4 | -0.01 | -0.08 | 0.05 | 0.06 | 0.13 | 0.14 | 0.16 | 0.08 | 0.21 | 1.00 |
| AZGP1 | 0.00 | -0.09 | 0.07 | 0.03 | 0.15 | 0.06 | 0.10 | 0.05 | 0.11 | 0.68 |
| C9 | -0.08 | -0.06 | -0.06 | -0.09 | 0.07 | 0.03 | -0.04 | -0.04 | -0.03 | 0.47 |
| CNDP1 | 0.14 | 0.12 | 0.11 | 0.19 | 0.22 | 0.25 | 0.31 | 0.20 | 0.27 | 0.46 |
| LGALS3BP | -0.24 | -0.23 | -0.13 | -0.16 | -0.06 | -0.10 | -0.10 | -0.12 | 0.01 | 0.38 |
| ORM1 | 0.09 | 0.02 | 0.06 | 0.02 | 0.10 | 0.12 | 0.01 | 0.06 | 0.10 | 0.45 |
| *AGE* | -0.15 | -0.21 | -0.16 | -0.14 | -0.16 | -0.12 | -0.13 | -0.13 | -0.10 | -0.02 |
| *ALT* | 0.05 | -0.01 | -0.04 | 0.00 | -0.03 | 0.12 | 0.03 | 0.07 | 0.01 | -0.09 |
| *BMI* | -0.17 | -0.15 | -0.16 | -0.13 | -0.17 | -0.02 | -0.07 | -0.05 | -0.04 | -0.04 |
| *Baseline HCV RNA* | -0.04 | -0.09 | -0.10 | -0.11 | -0.10 | -0.06 | -0.07 | -0.04 | -0.06 | -0.11 |
|  |  |  |  |  |  |  |  |  |  | |
|  | AZGP1 | C9 | CNDP1 | LGALS3BP | ORM1 | AGE | *ALT* | *BMI* | *Baseline viral titer* | |
| CLIC1 | 0.00 | -0.08 | 0.14 | -0.24 | 0.09 | -0.15 | 0.05 | -0.17 | -0.04 | |
| ZYX | -0.09 | -0.06 | 0.12 | -0.23 | 0.02 | -0.21 | -0.01 | -0.15 | -0.09 | |
| TAGLN2 | 0.07 | -0.06 | 0.11 | -0.13 | 0.06 | -0.16 | -0.04 | -0.16 | -0.10 | |
| PFN1 | 0.03 | -0.09 | 0.19 | -0.16 | 0.02 | -0.14 | 0.00 | -0.13 | -0.11 | |
| FERMT3 | 0.15 | 0.07 | 0.22 | -0.06 | 0.10 | -0.16 | -0.03 | -0.17 | -0.10 | |
| PARVA/B | 0.06 | 0.03 | 0.25 | -0.10 | 0.12 | -0.12 | 0.12 | -0.02 | -0.06 | |
| GSTP1 | 0.10 | -0.04 | 0.31 | -0.10 | 0.01 | -0.13 | 0.03 | -0.07 | -0.07 | |
| VCL | 0.05 | -0.04 | 0.20 | -0.12 | 0.06 | -0.13 | 0.07 | -0.05 | -0.04 | |
| TLN1 | 0.11 | -0.03 | 0.27 | 0.01 | 0.10 | -0.10 | 0.01 | -0.04 | -0.06 | |
| APOA4 | 0.68 | 0.47 | 0.46 | 0.38 | 0.45 | -0.02 | -0.09 | -0.04 | -0.11 | |
| AZGP1 | 1.00 | 0.61 | 0.42 | 0.11 | 0.48 | -0.13 | -0.16 | -0.14 | -0.17 | |
| C9 | 0.61 | 1.00 | 0.35 | -0.02 | 0.47 | -0.21 | -0.24 | -0.07 | -0.15 | |
| CNDP1 | 0.42 | 0.35 | 1.00 | -0.16 | 0.31 | -0.19 | -0.13 | 0.06 | -0.17 | |
| LGALS3BP | 0.11 | -0.02 | -0.16 | 1.00 | 0.14 | 0.11 | -0.02 | 0.01 | 0.01 | |
| ORM1 | 0.48 | 0.47 | 0.31 | 0.14 | 1.00 | -0.14 | -0.28 | 0.11 | -0.19 | |
| *AGE* | -0.13 | -0.21 | -0.19 | 0.11 | -0.14 | 1.00 | 0.16 | 0.16 | 0.26 | |
| *ALT* | -0.16 | -0.24 | -0.13 | -0.02 | -0.28 | 0.16 | 1.00 | 0.11 | 0.14 | |
| *BMI* | -0.14 | -0.07 | 0.06 | 0.01 | 0.11 | 0.16 | 0.11 | 1.00 | 0.13 | |
| *Baseline HCV RNA* | -0.17 | -0.15 | -0.17 | 0.01 | -0.19 | 0.26 | 0.14 | 0.13 | 1.00 | |
