# Supplementary material for: A signature for immune response correlates with HCV treatment outcome in Caucasian subjects
Source: Data Brief. 2015 Feb 11;3:56–61. doi: 10.1016/j.dib.2015.01.009 (PMC4510051; doi:10.1016/j.dib.2015.01.009)
Supplement: Supplementary file 1 — Supplementary data [file mmc1.zip › supp_table9.docx]

Supplementary Table 9: Data for Figure 4 in manuscript

| Regimen | Genotype | Treatment-Naïve SVR | Null Responder SVR | Reference |
| --- | --- | --- | --- | --- |
| Sovaldi+GS-9669+RBV | G1 | 92.0% (N=25) | 100.0% (N=10) | [2] |
| Sovaldi+GS-5885+RBV | G1 | 97.8% (N=456) | 100.0% (N=9) | [2] |
| Sovaldi+RBV | G1 | 70.0% (N=50) | 10.0% (N=10) | [10] |
| Sovaldi+RBV | G3 | 63.0% (N=176) | 27.0% (N=15) | [11] |
| Sovaldi+RBV, 12 wks | G2 | 95.0% (N=73) | 70.0% (N=10) | [11] |
| ABT-450/r+ ABT-333+RBV | G1 | 93.0% (N=14) | 50.0% (N=6) | [12] |
| ABT-450/r+ ABT-267+ ABT-333+RBV (12 wks) | G1 | 98.7% (N=79) | 93.3% (N=45) | [3] |
| ABT-450/r+ ABT-267+ ABT-333+RBV (24 wks) | G1 | 96.2% (N=80) | 97.7% (N=43) | [3] |
| PR | G1 | 49.3% (N=1356) | 9.4% (N=53) | [4] |
| Incivek+PR | G1 | 79.0% (N=363) | 29.0% (N=72) | [4] |
| Incivek+PR, 4wk PR lead-in | G1 | 79.0% (N=363) | 33.0% (N=75) | [4] |
| BI201335+PR, 3 day PR lead-in | G1 | 73.4% (N=143) | 20.4% (N=56) | [5],[6] |
| BI201335+PR | G1 | 83.3% (N=144) | 38.7% (N=38) | [5],[6] |
| OLYSIO+PR | G1 | 80.0% (N=521) | 53.0% (N=17) | [7] |
