# Supplementary figures and images for: A signature for immune response correlates with HCV treatment outcome in Caucasian subjects
Source: Data Brief. 2015 Feb 11;3:56–61. doi: 10.1016/j.dib.2015.01.009 (PMC4510051; doi:10.1016/j.dib.2015.01.009)

## Slide 1
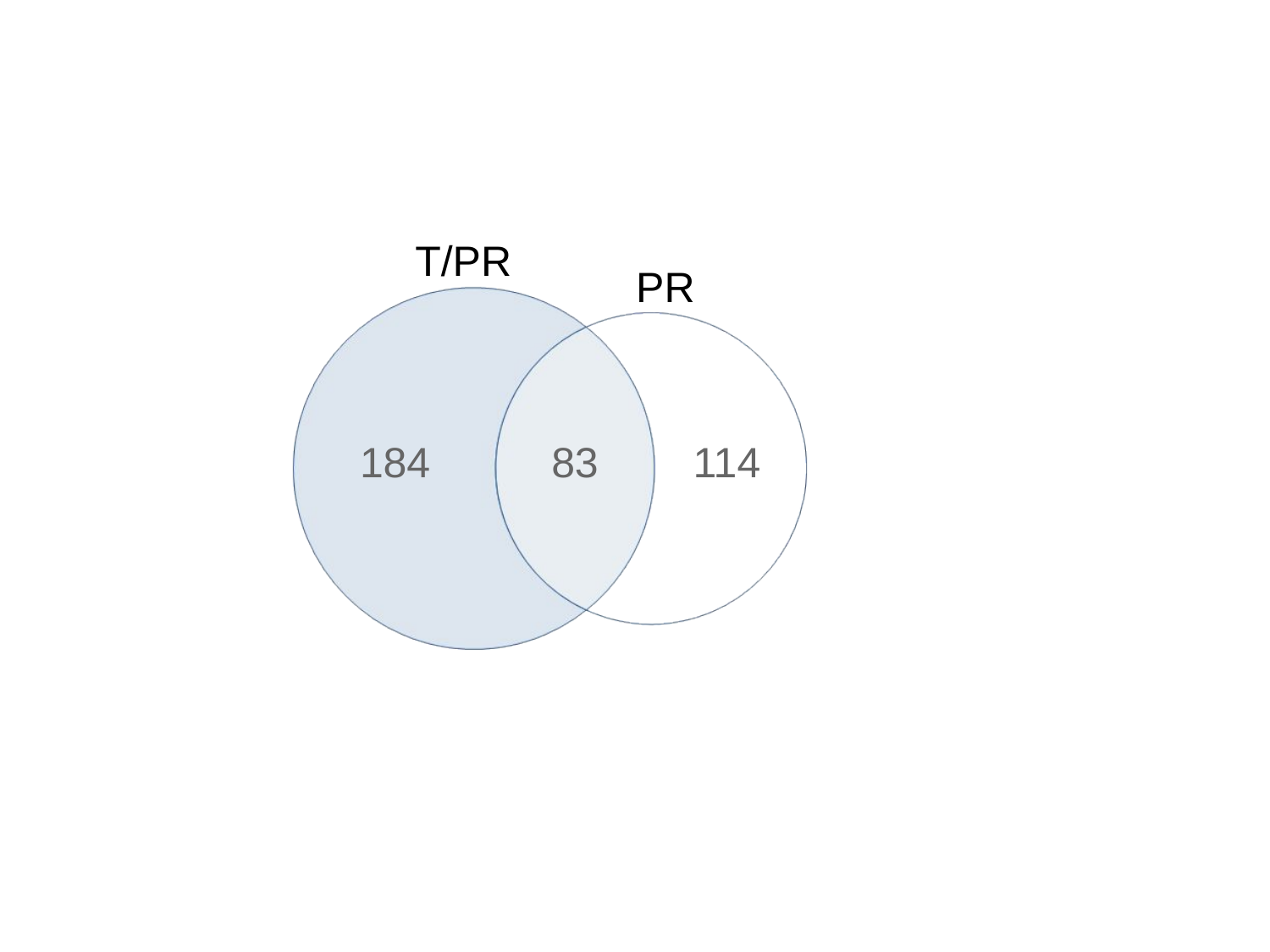

T/PR
PR
184
83
114

Supplement: Supplementary file 1 — Supplementary data [file mmc1.zip › supp_fig1.pptx]

## Slide 1
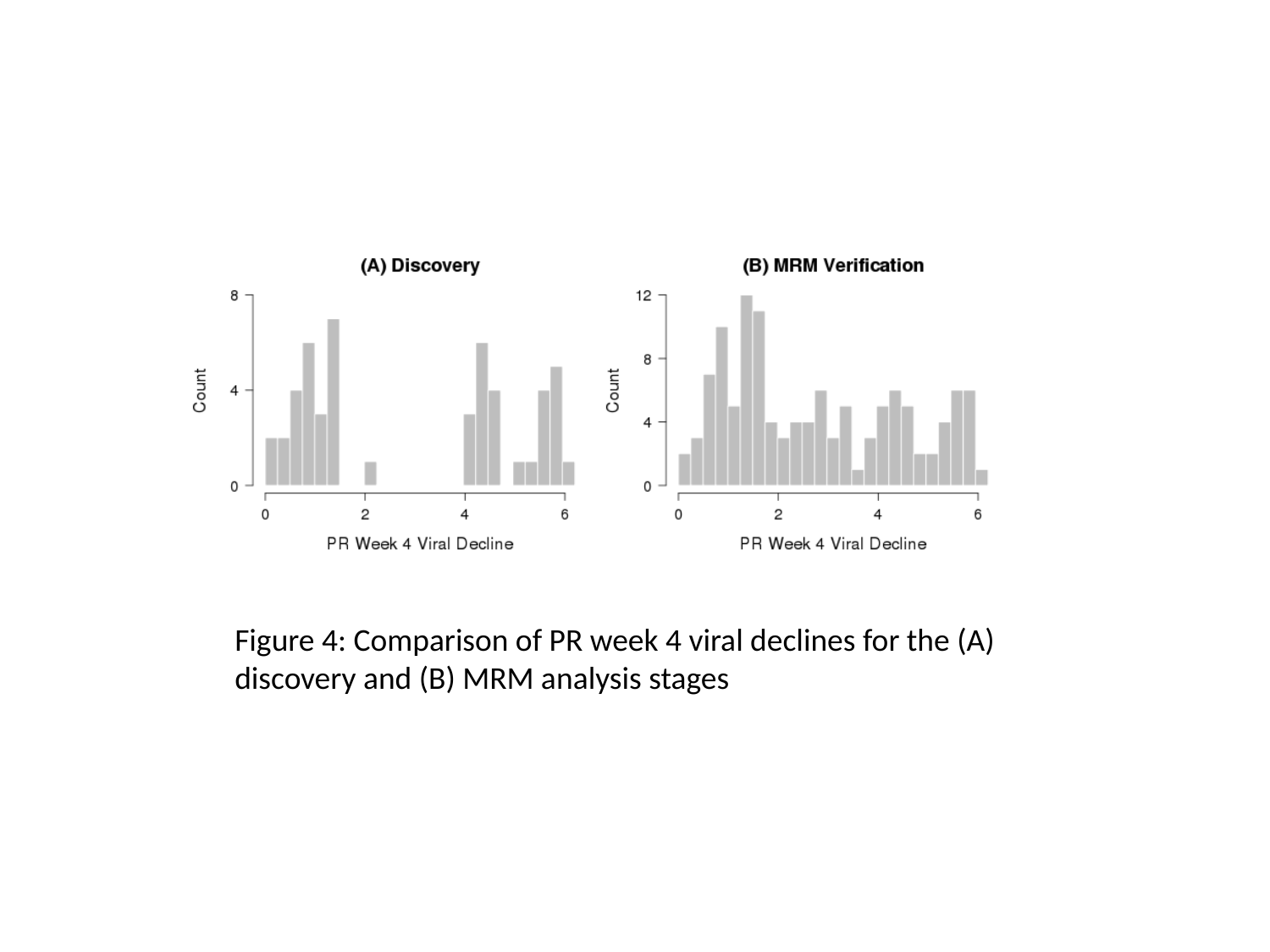

Figure 4: Comparison of PR week 4 viral declines for the (A) discovery and (B) MRM analysis stages

Supplement: Supplementary file 1 — Supplementary data [file mmc1.zip › supp_fig4.pptx]
